# Supplementary material for: Ultrahigh-fidelity spatial mode quantum gates in high-dimensional space by diffractive deep neural networks
Source: Light Sci Appl. 2024 Jan 5;13:10. doi: 10.1038/s41377-023-01336-7 (PMC10767004; doi:10.1038/s41377-023-01336-7)
Supplement: Supplementary file 1 — Supplementary Information [file 41377_2023_1336_MOESM1_ESM.pdf]

Supplementary Information for

Ultrahigh-Fidelity Spatial Mode Quantum Gates in High-Dimensional Space by Deep Diffractive Neural Networks

Qianke Wang[1,2,†], Jun Liu[1,2,†], Dawei Lyu[1,2], Jian Wang[1,2,*]

[1] *Wuhan National Laboratory for Optoelectronics and School of Optical and Electronic Information, Huazhong University of Science and Technology, Wuhan 430074, Hubei, China*

[2] *Optics Valley Laboratory, Wuhan 430074, Hubei, China*

[†] *These authors contributed equally to this work.*

[*] *Corresponding Author: jwang@hust.edu.cn*

Supplementary Note 1: Phase pattern generation

We employ the TensorFlow 2.5.0 architecture to model the physical diffraction layers and train the network models. Each layer is defined as a complex value matrix, which is multiplied by the incoming field as the modulation process. The angular spectrum method is used to describe the free-space propagation, and the mean squared error between the ideal output field and the inference is defined as the loss function. The D^2NN architecture is shown in Fig. S1. To compensate for systematic errors, we introduce random offsets and modulation blurring correction to the complex-valued layer model, which are discussed in detail in Supplementary Note 6 and Note 7. We use the Adam optimizer with default parameters in TensorFlow, except that the learning rate was set to 0.01. Adam combines the advantages of adaptive learning rates from RMSProp and the momentum from SGDM. It adapts the learning rates per parameter and includes a moving average of past gradients. Adam also adds bias-correction compared to RMSProp. The comparison of the accuracy versus training numbers of different optimizers are plotted in Fig. S2. It should be noted that this learning rate may not be optimal, and further exploration could improve network performance.

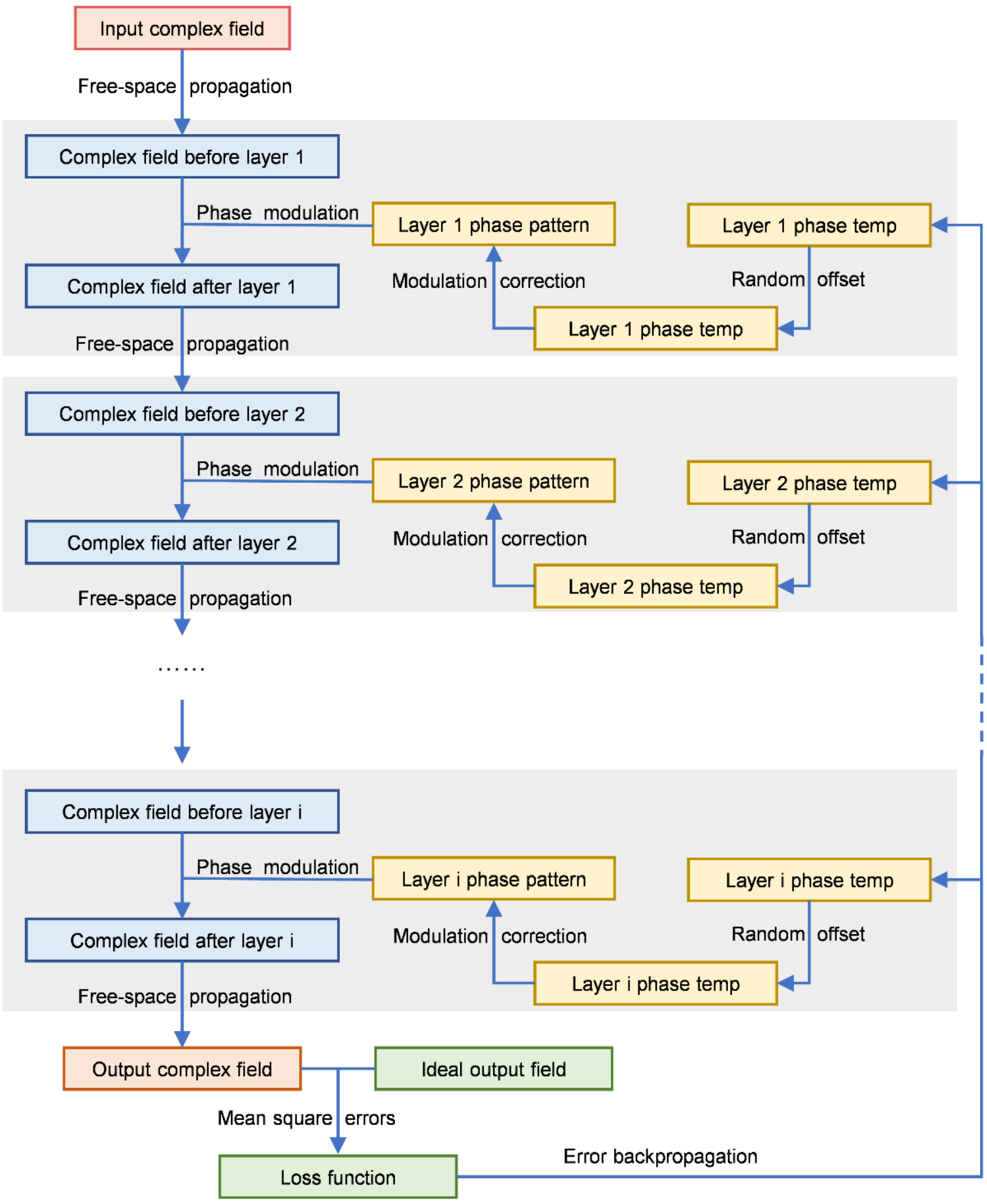

Fig. S1. Architecture of D^{2}NN using TensorFlow.

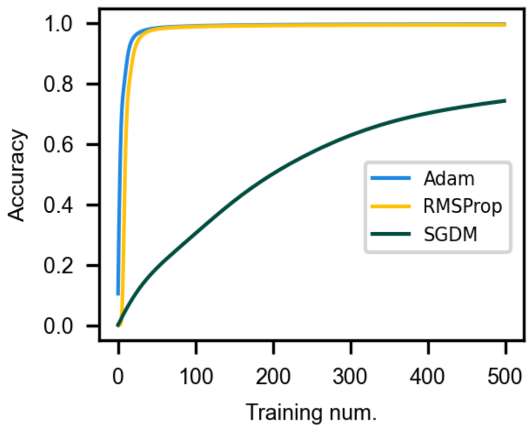

Fig. S2. Accuracy versus training number of SGDM (learning rate= 0.1, momentum=0.9), RMSProp (learning rate=

0.01), and Adam optimizer (learning rate= 0.01).

Supplementary Note 2: Matrix representation of quantum gates, MUBs and complement results

In the three-dimensional space, the Pauli X gate is no longer limited to flipping two computational bases, but rather cyclic shift-transformation of three bases as the matrix representation described in Eq. (S1). Operation X_0 is the identity matrix and shifts no input. X_1 and X_2 shift the input basis states once and twice, respectively.

$$X_0 = \begin{pmatrix} 1 & 0 & 0 \\ 0 & 1 & 0 \\ 0 & 0 & 1 \end{pmatrix}, X_1 = \begin{pmatrix} 0 & 1 & 0 \\ 0 & 0 & 1 \\ 1 & 0 & 0 \end{pmatrix}, X_2 = \begin{pmatrix} 0 & 0 & 1 \\ 1 & 0 & 0 \\ 0 & 1 & 0 \end{pmatrix} \tag{S1}$$

The matrix representation of three-dimensional Hadamard gates is described by eq. (S2), where $\omega = e^{2\pi i/3}$.

$$H_0 = \begin{pmatrix} 1 & 0 & 0 \\ 0 & 1 & 0 \\ 0 & 0 & 1 \end{pmatrix}, H_1 = \frac{1}{\sqrt{3}}\begin{pmatrix} 1 & 1 & 1 \\ 1 & \omega & \omega^2 \\ 1 & \omega^2 & \omega \end{pmatrix}, H_2 = \frac{1}{\sqrt{3}}\begin{pmatrix} 1 & 1 & 1 \\ \omega & \omega^2 & 1 \\ \omega & 1 & \omega^2 \end{pmatrix}, H_3 = \frac{1}{\sqrt{3}}\begin{pmatrix} 1 & 1 & 1 \\ \omega^2 & 1 & \omega \\ \omega^2 & \omega & 1 \end{pmatrix} \tag{S2}$$

According to ref. 1, all inequivalent sets of MUBs in three dimensions could be derived from the Hadamard matrices. The state vectors $|\psi_i\rangle$ are the columns of these Hadamard matrices, thus 12 states in total. It is convenient to verify by calculating $|\langle\psi_i|\psi_j\rangle|^2 = 1/d$, where d is the dimension.

The *CNOT* gate operating on 2×2-dimensional space has more complicated MUBs. We construct an over-complete state set from the column vectors in matrix S which is the tensor product of six two-dimensional MUBs, as shown in eq. (S3). This state set with 36 state vectors oversteps the range of four-dimensional MUBs but still works in quantum process tomography.

$$\begin{gathered} C = \begin{pmatrix} 1 & 0 & \frac{1}{\sqrt{2}} & \frac{1}{\sqrt{2}} & \frac{1}{\sqrt{2}} & \frac{1}{\sqrt{2}} \\ 0 & 1 & \frac{1}{\sqrt{2}} & \frac{-1}{\sqrt{2}} & \frac{i}{\sqrt{2}} & \frac{-i}{\sqrt{2}} \end{pmatrix} \\ S = C \otimes C \end{gathered} \tag{S3}$$

As a complement to the results presented in the main text, we characterize the rest of the three-dimensional gates, including the X_2, H_2, and H_3 gates. Fig. S3a-c presents the tomography matrices of these three gates, and Fig. S3d-f are the corresponding reconstructed process matrices χ. The fidelities are around 97% revealing decent performance.

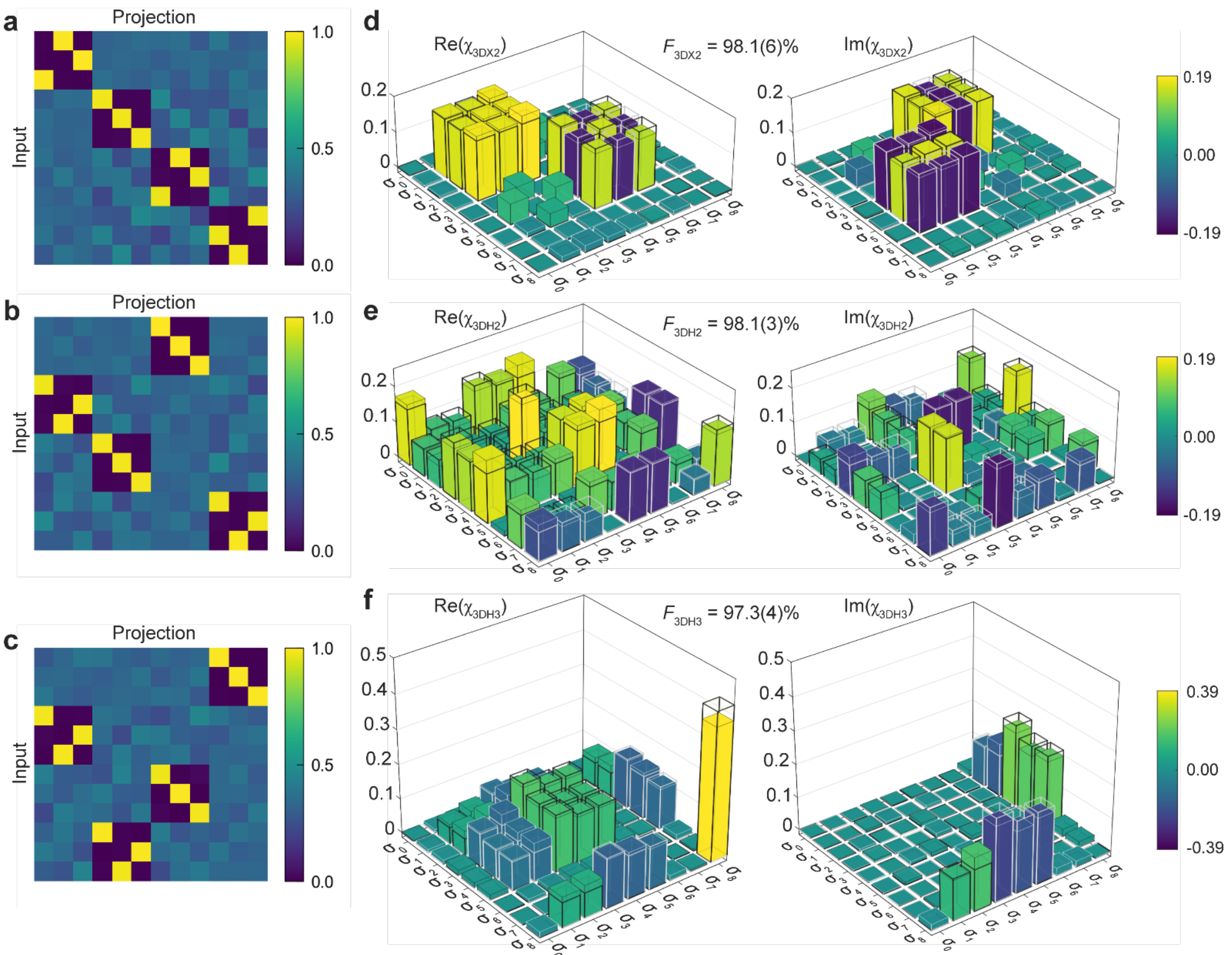

Fig. S3. Quantum process tomography of spatial mode quantum gates. a-c Tomography matrices and **d-f** reconstructed process matrices χ for the X_2 gate, H_2 gate and H_3 gate.

Supplementary Note 3: Quantum process tomography reconstruction with maximum-likelihood-estimation method

The maximum-likelihood-estimation principle as a mature method has been applied in quantum state tomography and quantum process tomography for a long time. Here we briefly introduce the procedure of it. Considering an unknown quantum process E is a linear completely positive map M_E from the Hilbert space H to the Hilbert space K, we can test it with a bunch of probe states ρ_m, and the output states $\rho_{out} = M_E(\rho_m) = \mathrm{Tr}_H\left(E\rho_m{}^T \otimes I_K\right)$, where Tr_H is the partial trace of space H, and I_K is the identity operator on space K, and T denotes the transposition. The trace-preserve (TP) condition leads to $\mathrm{Tr}_K(\rho_{out}) = \mathrm{Tr}_H(\rho_m)$ for any ρ_m, and the operator E must satisfy

$$\mathrm{Tr}_K\left(E\right)=I_H \tag{S4}$$

where I_H is the identity operator on space H. Measurements Π_n are carried out on the corresponding output states, yielding theoretical probabilities $p_{mn}=\mathrm{Tr}\left(\rho_{out}\Pi_n\right)=\mathrm{Tr}\left(E\rho_m{}^T\otimes\Pi_n\right)$. Let f_{mn} denote the experimental frequency of detecting ρ_m with Π_n, and the estimated operator E should maximize the log-likelihood function

$$L\left(f_{mn},p_{mn}\left(E\right)\right)=\sum_{m,n}f_{mn}\ln p_{mn}-\mathrm{Tr}\left(\Lambda E\right) \tag{S5}$$

where $\Lambda=\lambda\otimes I_K$, and λ is the Lagrange multiplier in matrix form to satisfy the TP condition of Eq. (S4). Varying Eq. (S5) with respect to E will give the extremal equation for operator E

$$\begin{aligned}L\left(f_{mn},p_{mn}\left(E+\delta E\right)\right)-L\left(f_{mn},p_{mn}\left(E\right)\right)=0\\ \mathrm{Tr}\left(\left(\sum_{m,n}\frac{f_{mn}}{p_{mn}}\rho_m{}^T\otimes\Pi_n-\Lambda\right)\delta E\right)=0\end{aligned} \tag{S6}$$

Eq. (S6) holds for all δE, then

$$\begin{aligned}&R=\Lambda,\ (R=\sum_{m,n}\frac{f_{mn}}{p_{mn}}\rho_m{}^T\otimes\Pi_n)\\ &\Lambda^{-1}RE=E,\ \text{or}\\ &ER\Lambda^{-1}=E\end{aligned} \tag{S7}$$

Combining two variations of Eq. (S7), the symmetrical expression suitable for iterations is derived

$$E_{i+1}=\Lambda_i^{-1}R_iE_iR_i\Lambda_i^{-1},\ \lambda_i=\left(\mathrm{Tr}_K\left(R_iE_iR_i\right)\right)^{\frac{1}{2}} \tag{S8}$$

where i denotes the i-th iteration. This expression preserves the positive semi-definiteness and trace normalization of the operator since $R_iE_iR_i$ is positive-definite and the iterations satisfy $\mathrm{Tr}_K\left(E\right)=I_H$. Let $E_0=I_{H\otimes K}/d_K$, where d_K is the dimension of space K, the estimated operator E_i will gradually approach the theoretical operator of the test results during the numerical iterations.

Supplementary Note 4: Example of the Deutsche algorithm

As can be seen from Fig. S4, the Deutsche algorithm consists of four steps:

1. Prepare input states. The first qubit labeled as x is initialized to $|0\rangle_x$, and the second qubit y is initialized to $|0\rangle_y$. Then apply H gates to each qubit, yielding

$$|\psi_1\rangle = \frac{|0\rangle_x + |1\rangle_x}{\sqrt{2}} \otimes \frac{|0\rangle_y - |1\rangle_y}{\sqrt{2}} \tag{S9}$$

2. Apply the quantum oracle. The oracle maps the input state $|x\rangle|y\rangle$ to $|x\rangle|y \oplus f(x)\rangle$, thus the output is

$$\begin{aligned}|\psi_2\rangle &= \frac{|0\rangle_x + |1\rangle_x}{\sqrt{2}} \otimes \frac{|0\rangle_y \oplus f(x) - |1\rangle_y \oplus f(x)}{\sqrt{2}} \\ &= (-1)^{f(x)} \frac{|0\rangle_x + |1\rangle_x}{\sqrt{2}} \otimes \frac{|0\rangle_y - |1\rangle_y}{\sqrt{2}}\end{aligned} \tag{S10}$$

When the qubit x is $|0\rangle_x$, the corresponding $f(x)$ is $f(0)$, and vice versa. Note the x and y used in the Dirac notation only indicate the qubits and do not have any assigned value, whereas the x and y used as inputs in the oracle have assigned values. So, the Eq. (S10) could be

$$|\psi_2\rangle = \frac{(-1)^{f(0)}|0\rangle_x + (-1)^{f(1)}|1\rangle_x}{\sqrt{2}} \otimes \frac{|0\rangle_y - |1\rangle_y}{\sqrt{2}} \tag{S11}$$

3. Apply an H gate to each qubit. The final state will be

$$|\psi_3\rangle = |f(0) \oplus f(1)\rangle_x \otimes |1\rangle_y = \begin{cases} |0\rangle_x \otimes |1\rangle_y, & f(x) \text{ is constant} \\ |1\rangle_x \otimes |1\rangle_y, & f(x) \text{ is balanced} \end{cases} \tag{S12}$$

4. Measure the final state $|\psi_3\rangle$. Theoretically, only the measurement of qubit x is necessary, and a constant function will be measured as $|0\rangle_x$ and a balanced function as $|1\rangle_x$.

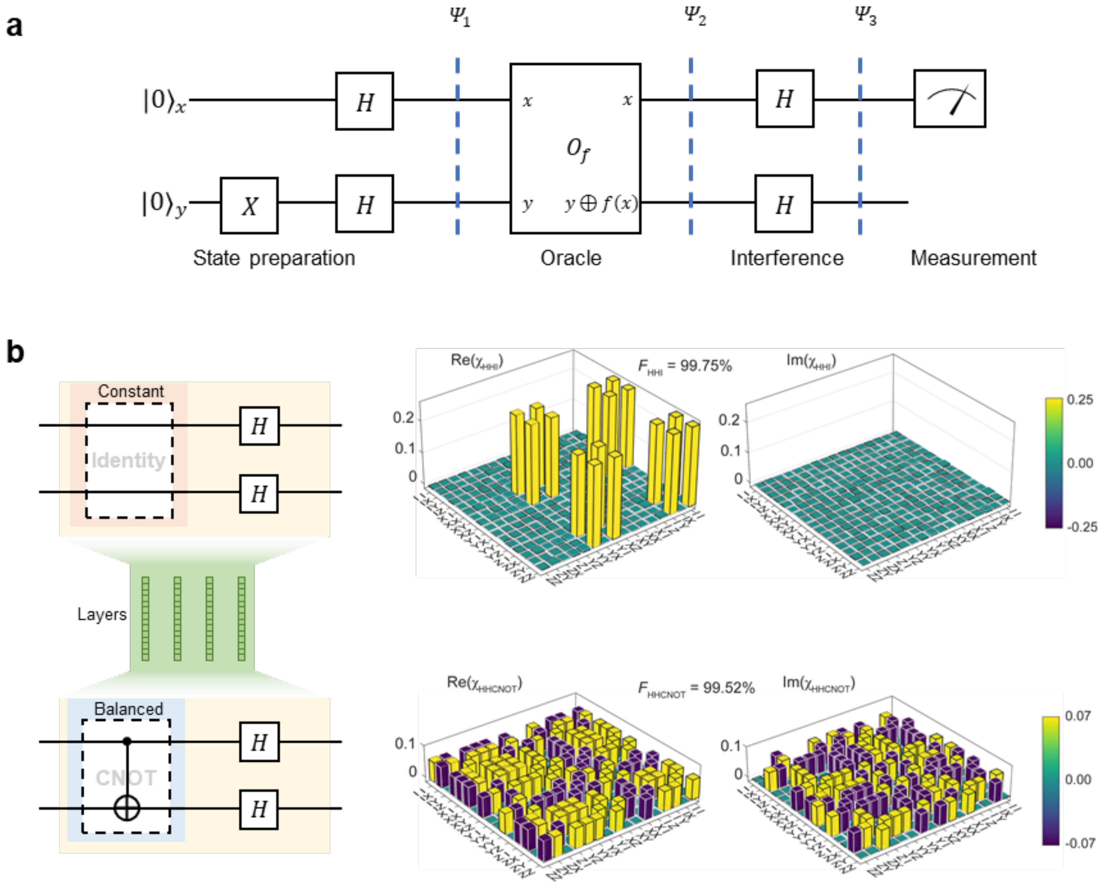

Fig. S4. Deutsch algorithm diagrams. a Quantum circuit for the Deutsch algorithm. The circuit can be subdivided into four main components. The oracle function denoted as O_f applies identity operator to qubit x, while applying XOR with $f(x)$ to qubit y. **b** Combinations of the Oracle operations and interference operations in a single D^{2}NN and corresponding process matrices.

The $CNOT$ gate is an example that implements a balanced function:

$$
\begin{aligned}
CNOT\left(\frac{|0\rangle_x+|1\rangle_x}{\sqrt{2}}\otimes\frac{|0\rangle_y-|1\rangle_y}{\sqrt{2}}\right) &= CNOT\frac{1}{2}\left(|0\rangle_x+|1\rangle_x\right)\left(|0\rangle_y-|1\rangle_y\right)\\
&=\frac{1}{2}|0\rangle_x\left(|0\rangle_y\oplus f(0)-|1\rangle_y\oplus f(0)\right)\\
&+\frac{1}{2}|1\rangle_x\left(|0\rangle_y\oplus f(1)-|1\rangle_y\oplus f(1)\right)\\
&=\frac{1}{2}\left(|0\rangle_x-|1\rangle_x\right)\left(|0\rangle_y-|1\rangle_y\right)\\
&\xrightarrow{H\otimes H}=\frac{1}{2}|1\rangle_x|1\rangle_y
\end{aligned}
\tag{S13}
$$

Similarly, the identity operation is an example of a constant function implementation:

$$
\begin{aligned}
I\left(\frac{|0\rangle_x+|1\rangle_x}{\sqrt{2}}\otimes\frac{|0\rangle_y-|1\rangle_y}{\sqrt{2}}\right) &= I\frac{1}{2}\left(|0\rangle_x+|1\rangle_x\right)\left(|0\rangle_y-|1\rangle_y\right)\\
&=\frac{1}{2}\left(|0\rangle_x+|1\rangle_x\right)\left(|0\rangle_y-|1\rangle_y\right)\\
&\xrightarrow{H\otimes H}=\frac{1}{2}|0\rangle_x|1\rangle_y
\end{aligned}
\tag{S14}
$$

The characteristic of implementing a simple combination of multiple basic quantum gates in a single D^{2}NN is also showcased through the application of the Deutsch algorithm. In essence, the D^{2}NN functions by fitting the transformation matrix of the inputs and outputs. For a given number of dimensions, such as the case with four dimensions here, a combination of multiple basic quantum gates still results in a four-dimensional unitary matrix, thereby maintaining the same fitting complexity for the D^{2}NN. To provide specific details, the gate loaded onto SLM2 is represented as $(H\otimes H)I_4$ for the constant situation and $(H\otimes H)CNOT$ for the balanced situation. Here, H signifies the two-dimensional Hadamard gate, and I_4 represents the four-dimensional identity matrix. The process matrices for these two situations are visually presented in Fig. S4b. Additionally, the compression of a more extensive two-qubit circuit or higher dimensions, are also possible.

Supplementary Note 5: Update rules for spacing optimization

Finding the optimal spacing that maximizes performance is an optimization problem that is difficult to solve for physical systems. Fortunately, in our case, finding a local optimum is sufficient. To achieve this, we use a

search protocol that scans a given range with a certain precision. The pseudo-code for the protocol is as follows.

Algorithm 1: Our update rules for spacing optimization. Function $\mathrm{idx}(x)$ gives the index of x in an array. Function $\mathrm{V}(s)$ gives the output state visibility of the spacing s

---

Require: $\{R_{e,n}\}$, $n \in \{0,1\}$, $R_{e,0} < R_{e,1}$: Estimated searching range

Require: $\{R_{T,n}\}$, $n \in \{0,1\}$, $R_{T,0} < R_{T,1}$: Searching range threshold

Require: s_T : Spacing threshold

$\{R_{0,n}\} \leftarrow \{R_{e,n}\}$ (Initialize searching range)

$i \leftarrow 0$ (Initialize step number)

while $R_{T,0} < s_{i,m} < R_{T,1}$ && $s > s_T$, $(m \in \{0,1,2,3,4\})$

  $s_{i,m} \leftarrow R_{i,0} + m \cdot (R_{i,1} - R_{i,0}) / 4$

  if $\mathrm{idx}(\min(\mathrm{V}(s_{i,m}))) == 0$ && $\mathrm{idx}(\max(\mathrm{V}(s_{i,m}))) == 4$

    $R_{i,0} \leftarrow s_{i,0}$

    $R_{i,1} \leftarrow s_{i,0} + 2 \cdot (s_{i,4} - s_{i,0})$

  else if $\mathrm{idx}(\min(\mathrm{V}(s_{i,m}))) == 4$ && $\mathrm{idx}(\max(\mathrm{V}(s_{i,m}))) == 0$

    $R_{i,0} \leftarrow s_{i,4} - 2 \cdot (s_{i,0} - s_{i,4})$

    $R_{i,1} \leftarrow s_{i,4}$

  else if $0 < \mathrm{idx}(\max(\mathrm{V}(s_{i,m}))) < 4$

    $d \leftarrow \mathrm{idx}(\max(\mathrm{V}(s_{i,m})))$

    $R_{i,0} \leftarrow s_{i,d-1}$

    $R_{i,1} \leftarrow s_{i,d+1}$

  end if

  $i \leftarrow i + 1$

end while

Supplementary Note 6: Misalignment analysis

Misalignment is an inevitable factor that affects the experiment. To mitigate its impact, it is important to understand different types of misalignment. In our experiment, we observe offsets that usually arise in the direction perpendicular (Z-axis) and parallel (X-axis) to the layers, as shown in the upper left legend of Fig. S5a. The Y-axis offset behavior is expected to be similar to the X-axis and is therefore neglected. To evaluate the degradation caused by misalignment, we plot the simulated visibility and energy loss of the output states and plot the field profiles for better visualization. Fig. S5a-b show intuitive results and lager offsets contribute to worse performance. It can be seen from this figure that the tolerances to both kinds of offsets are very small. To enhance the robustness of the system, we introduce a spacing optimization process (described in the main text) to address Z-axis misalignment, and a random offset method to address X-axis misalignment.

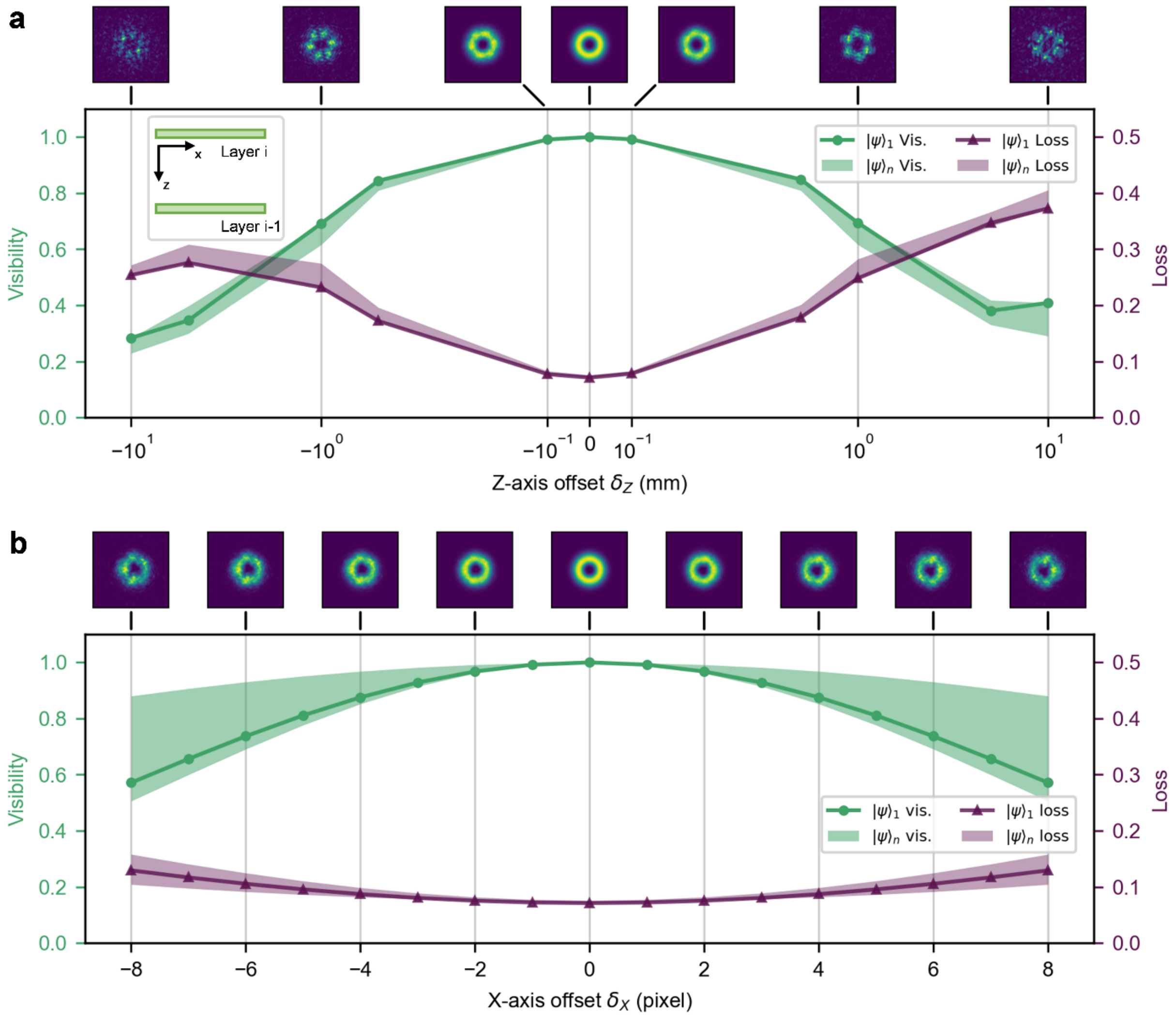

Fig. S5. Degradation of output states due to misalignment. The visibilities and energy losses of output states with different **a** Z-axis offsets δ_Z and **b** X-axis offsets δ_X. The upper left legend in **a** provides the definition of the axes. The profiles of output state $|\psi_1\rangle$ are displayed at the top of each plot. The solid lines show the visibilities and losses of $|\psi_1\rangle$, and the filled areas represent the range of all output states $|\psi_n\rangle$.

The random x-offset follows a Gaussian distribution, and the performance of D^2NN as a function of the distribution parameter σ is shown in Fig. S6a. Larger values of σ, which indicate greater random shifts of the layers during the training process, result in deteriorated visibilities and energy losses of the output states. Fig. S6b visually illustrates the effect of different σ values on the generated layers and corresponding output states. As σ increases, the phase layers become smoother, leading to better tolerance to offsets but worse output state quality. These results suggest there is a trade-off between parameter σ and the offset tolerance. Based on this analysis, we choose a value of σ (0.3) in our demonstration that improves the smoothness of the layers while maintaining acceptable output quality.

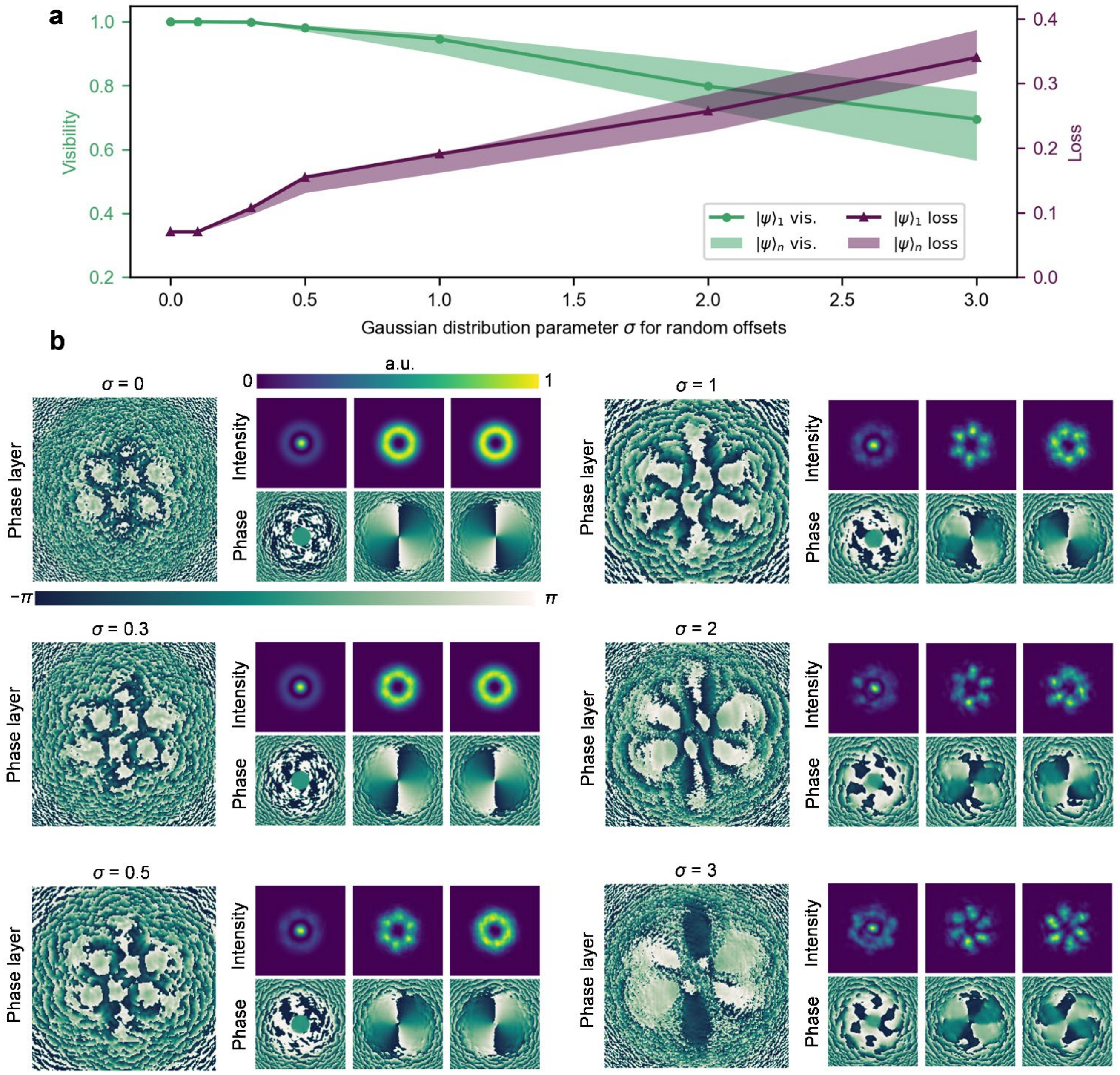

Fig. S6. Training process with random offsets to enhance misalignment tolerance. a The visibilities and energy losses of output states with different Gaussian distribution parameters σ for random offsets. **b** Visualization of generated layers and output states of computational basis corresponding to different σ. Larger σ, which means higher random offset level, leads to smoother phase layers and more robustness to offsets, at the cost of the output state quality (lower visibility and higher loss).

Supplementary Note 7: Modulation correction

The ideal modulation of phase wraps should be as sharp as the green curve plotted in Fig. S7a. However, in reality, modulation devices are subject to finite spatial resolution, manufacturing imperfections, and fringing fields between pixels, resulting in modulation blurring at the phase wraps, known as the fringe effect. This phenomenon can introduce extra scatter and damage to performance, particularly when the phase layers of D^2NN have many phase wraps as illustrated in Fig. S7b. To mitigate this degradation, one straightforward method is to include it in our D^2NN model. Leveraging the flexibility of D^2NN, we multiply the blurring function on each layer model as a correction so that the effect of blurring is also considered during the training process. As can be seen from Fig. S7c, the generated phase layer with correction exhibits lower spatial frequencies. The phase layers used in the experiment are all after correction and have helped improve experimental performance.

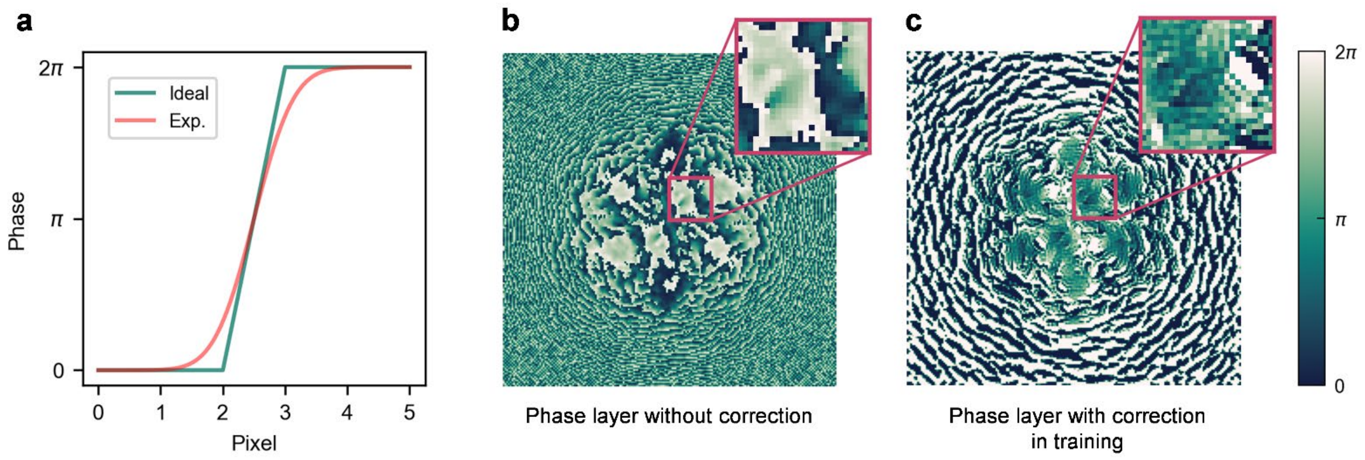

Fig. S7. Modulation correction of the phase layers. a The SLM modulation blurring between pixels during phase wraps of 0 and 2π . **b** A typical phase layer generated without modulation correction. **c** The phase layer generated with modulation correction in the training process. In comparison, the training process with modulation correction can significantly reduce phase wraps and improve the smoothness of the phase layers.

Reference

1. Brierley, S., Weigert, S. & Bengtsson, I. All mutually unbiased bases in dimensions two to five. *Quantum Inf. Comput.* **10**, 803–820 (2010).
