# Supplementary figures and images for: Ultrahigh-fidelity spatial mode quantum gates in high-dimensional space by diffractive deep neural networks
Source: Light Sci Appl. 2024 Jan 5;13:10. doi: 10.1038/s41377-023-01336-7 (PMC10767004; doi:10.1038/s41377-023-01336-7)

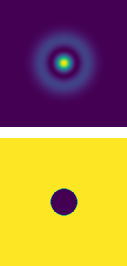

Supplement: Supplementary file 2 — propagation_3DX [file 41377_2023_1336_MOESM2_ESM.gif]
